# Role of parasitic vaccines in integrated control of parasitic diseases in livestock

**DOI:** 10.14202/vetworld.2015.590-598

**Published:** 2015-05-14

**Authors:** Neelu Sharma, Veer Singh, K. P. Shyma

**Affiliations:** Department of Veterinary Parasitology, College of Veterinary Science and Animal Husbandry, Sardarkrushinagar Dantiwada Agricultural University, Sardarkrushinagar - 385 506, Gujarat, India

**Keywords:** drug resistance, integrated control measures, parasitic infections, parasitic vaccines

## Abstract

Parasitic infections adversely affect animal’s health and threaten profitable animal production, thus affecting the economy of our country. These infections also play a major role in the spread of zoonotic diseases. Parasitic infections cause severe morbidity and mortality in animals especially those affecting the gastrointestinal system and thus affect the economy of livestock owner by decreasing the ability of the farmer to produce economically useful animal products. Due to all these reasons proper control of parasitic infection is critically important for sustained animal production. The most common and regularly used method to control parasitic infection is chemotherapy, which is very effective but has several disadvantages like drug resistance and drug residues. Integrated approaches to control parasitic infections should be formulated including grazing management, biological control, genetic resistance of hosts, and parasitic vaccines. India ranks first in cattle and buffalo population, but the majority of livestock owners have fewer herds, so other measures like grazing management, biological control, genetic resistance of hosts are not much practical to use. The most sustainable and economical approach to control parasitic infection in our country is to vaccinate animals, although vaccines increase the initial cost, but the immunity offered by the vaccine are long lived. Thus, vaccination of animals for various clinical, chronic, subclinical parasitic infections will be a cheaper and effective alternative to control parasitic infection for long time and improve animal production.

## Introduction

Infections caused by parasites are responsible for most devastating and prevalent diseases in humans and animals. Parasitic infections are related with huge economic losses in terms of production losses, losses due to infertility. Control of parasitic diseases in animals should be aimed to increase the productivity and body weight. Therefore, efficient, economic, and sustainable control methods are to be used. Despite the availability of a number of effective drugs for the treatment of most important diseases, a pressing need for the development of successful vaccines remains unexplored. The reasons for the development of vaccines is due to the increasing problem of resistance of parasite, lack of newly developed effective drugs, and the presence of drug residues in milk, milk derived products, and meat.

Hence, to control parasitic diseases a multidisciplinary approach involving the integration of chemotherapy, grazing management, biological control, genetic resistance of hosts, and parasitic vaccines should be implemented. Many parasites possess sophisticated immune evasion mechanisms making it at present difficult to conceive the development of efficient vaccines. However, the rapid development of immunology and genetic manipulation of cells will perhaps change these prospective in near future.

## Parasitic Infections and Economic Loss in Animal Production in India

During the last 5 years incidence of gastrointestinal (GI) parasitic infections reported in India ranged between 25% and 78% [1]. The most commonly reported GI parasitic infections include: *Haemonchus contortus*, *Trichosrtongylus colubriformis*, *Paramphistomes*, *Fasciola gigantica*, *Strongyles* spp.*, Chabertia* spp., *Dicrocellium* spp. *Nematodirus helvatianus*, *Bunostomum trigonocephalum*, *Toxocara vitulorum*, *Moniezia* spp., *Trichuris* spp., *Strongyloides* spp., *Eimeria* spp., and *Balantidium* spp.

Economic losses due to GI parasitism in Rajasthan were Rs. 97.37 crores in adult and Rs. 21.79 crores in yearling sheep. A study from Uttar Pradesh showed that 4-18% increase in milk production was achieved following anthelmintic treatment. In a study from West Bengal, net economic loss of Rs. 195.33/sheep has been recorded due to GI nematodosis [2].

## Strategies to Control Parasitic Infections in Animals

Parasite control measures should be framed with the objectives of maintaining economic stability, sustainability, and their efficiency to control the parasite load. An integrated approach to control parasitic infections in animals consist of grazing management, breeding animals for genetic resistance against parasites, biological control, strategic chemotherapy, prophylactic vaccination [2].

### Grazing management

Grazing management is defined as “manipulation of animal grazing practice to achieve the optimum results by reducing the parasite burden [3,4].” In grazing animals, main source of spread of parasitic infection is through grazing of pastures containing parasite eggs and larvae and its decontamination is very difficult. The best ways to prevent pasture infection are:

- Controlled rotational grazing or pasture rotation system: It is a management technique involving the subdivision of pasteur, grazing of each paddock, and allows it to rest for time [5].
- Alternate or mixed grazing: In this system two or more host species are allowed to graze together in the same field, as in any given environment two different host species do not share common parasite species. This alteration between species can be a successful tool of management.
- Pasteur resting: Animals should not be allowed to graze in the same paddock for a longer time and preventing animals to do so is called as pasteur resting. Resting period varies from 2 months to 6 months depending upon climate. Studies conducted in semi-arid regions of Rajasthan revealed that sheep grazed during monsoon on spring contaminated, summer ungrazed pasture had very low fecal egg counts, pasture larval burden, and worm counts compared to those on continuously grazed contaminated pasture [6].
- Zero grazing: It is an alternative system in which livestock are housed and are fed with treated forage or silage that does not allow parasite growth. This approach carry a low risk of parasitic disease requiring no control measures and is an efficient system of energy conservation and use of land resources [2].

### Breeding animals for genetic resistance against parasites

Genetic resistance is best approach to sustainable parasite control. In Northern Australia, *Bos taurus* has been replaced by *Bos indicus* cattle as they are resistant to cattle tick and babesiosis. Nowaday’s research is going on the selection of breeds that are genetically resistant for GI nematode infections for both sheep and cattle [7,8]. There are over 30 indigenous breeds of cattle in India (e.g.,: Rathi, Gir, Kankrej, Tharparkar, Sahiwal, Deoni, Halliker, and Haryana) and 10 breeds of buffaloes (e.g.,: Murrah, Jaffarabadi, Mehsana, and Surti). All these breeds show resistance to various diseases including GI parasites [2]. Hence, selective cross breeding should be promoted to develop breeds that are genetically resistant to ticks and parasitic infections.

### Biological control

Larsen [9] has stated that nematophagous fungi such as *Duddingtonia flagrans*, *Drechmeria coniospora*, *Harposporium anguillulae*, *Verticillium chlamydosporium*, *Arthrobotrys oligospora*, *Paecilomyces lilacinus* naturally occur in feces of various ruminants. These fungi have the ability to infect and damage parasite eggs thus preventing the development of egg to infective larvae on pasture. As per Sanyal *et al*. [10], feeding of 5,00,000 spores of the fungi/kg body weight of the cattle or sheep as a bolus can significantly reduce the parasite burden. These fungi are very efficient in controlling *Haemonchus* spp., *Trichostrongylus* spp., *Ascaris* spp., *Fasciola* spp., and *Ostertagia* spp. of parasites.

Biological control of mosquitoes acting as vector for many diseases can be done by using Gambusia and Poeicila fishes. *Bacillus thuringiensis* israelensis (Bti) and *Bacillus sphaericus* (Bs) are also used extensively as biological control agent against mosquito larvae.

### Strategic chemotherapy

Chemotherapy is one of the most economical and effective and widely used approach to control parasitic infection in livestock. Several broad spectrum anthelmintics are currently available to control GI parasitic infection. Administering the therapeutic dose of anthelmintics during the peak season of parasitic infections can reduce the cost and maximize the benefit. Anthelmintic treatment can increase the productivity (>20%) and result in a significant economic gain (Rs. 46 million) in milking cows [1].

Despite the great impact of anthelmintic therapy on animal production, there are several disadvantages. One of the major being drug resistance. Resistance to anthelmintic agents has been reported among all the species of GI parasites in all parts of the world including India [11]. Indiscriminate and over use of anthelmintics is supposed to be the cause of this problem. Resistance can be prevented by the following strategies, i.e., strategic use of chemotherapy, “parasites in refugia” in grazing fields. “Parasites in refugia” means worm populations that are not exposed to anthelmintic treatment and are left to develop on pasteur [12]. Resistance can also be checked out by discovering drugs that have better and narrow spectrum of activity for parasites. Chemical residues in meat and milk are also a major disadvantage of using chemotherapy for control of parasitic diseases.

To overcome these problems narrow spectrum drugs should be discovered and chemicals that are rapidly degraded into harmless metabolites should be used. This will help in reduction of drug residues in animal food [13].

### Prophylactic vaccination

Vaccines are most efficient, reliable and sustainable method, and therefore also referred as green solutions to control parasitic disease and are useful in reducing reliance on pharmacological drugs and pesticides [2]. The use of vaccines has multiple benefits such as improving animal health and welfare by controlling animal infections; improving public health by controlling zoonoses and foodborne pathogens in animals; solving problems associated with resistance to acaricides, antibiotics, and anthelmintics; keeping animals and the environment free of chemical residues and maintaining biodiversity. All of these attributes should lead to improved sustainability of animal production and increasing economic benefit [14]. Descriptions of various parasitic vaccines are summarized below.

## Different Parasitic Vaccines

### Protozoan vaccines

Protozoan infections in animals are related to significant production losses and many organisms are also responsible for zoonotic diseases or have close relationships to human parasites, thus increasing their significance as infection reservoirs for human diseases. Vaccines for human protozoa are not available yet, but a number of veterinary vaccines are being formulated and are available [15] types of vaccines developed are:

### Live protozoan vaccines

Live vaccines are based on live organisms that stimulate an immune reaction in the hosts, mimicking natural infections. In protozoan infections, the immunological mechanisms involved in protection and the stages of protozoan parasite involved in infections are mostly not have been defined therefore, most of the vaccines make use of the live organisms itself to induce the required protective immune response. A live vaccination approach induces T-cell mediated immune responses through correct intracellular processing and presentation of antigens in association with major histocompatibility complex Class I and Class II antigens. T-cell responses are considered critical to protect against intracellular pathogens and live vaccination approaches more closely mimic the induction of both the innate and adaptive immune responses that would occur in natural infection and will therefore induce appropriate inflammatory and regulatory immune responses in the host animals [14]. The haemoprotozoans display antigenic diversity in their different life cycle stages within the host as well as between different species and strains and, within the same life cycle stages due to which vaccine development for these organisms is tough [16]. These also have a high degree of genetic complexity. Depending upon the format of infection different types of live vaccines are:

a) Vaccine based on complete but shortened life cycle infection (precocious strains of *Eimeria*)

Coccidiosis is caused by obligate intracellular protozoan parasite. These parasites undergoes a definite asexual cycles of merozoite production in the gut epithelial cells (3-4 cycles) before the production of infective stages i.e., oocysts. As the infection is self-limiting, therefore vaccination with a small dose of infective oocyst provides solid immunity against homologous infections with minimal pathology [15]. All the vaccines present in world are based on live virulent and live attenuated formulations. The process of attenuation has been achieved by several passages in chicken embryos or by using the precocious lines i.e., oocysts selected from naturally occurring “precocious” *Eimeria* strains that produce less merogenic cycles and these are safer to use. The early oocyst is found to be less pathogenic because of the decreased schizont size and lesser schizogonic stages. First commercial vaccine to fight with coccidiosis in poultry was Coccivax launched in USA 1951. This vaccine contains oocysts of *Eimeria acervulina*, *Eimeria maxima*, and *Eimeria mitis* and was developed for broilers. Live virulent vaccines available are Coccivac B (Merk Animal Health and is prepared using *E*. *acervullina*, *E*. *maxima*, *Eimeria tenella*, *Eimeria mivati*) Coccivac D, VAC M, ADVENT and inovocox registered in the USA; Immunocox C1 and Immunocox C2 registered in Canada and Nobilis Cox ATM from Netherlands. The live attenuated vaccines include *Eimeria* Vac (China); *Eimeria* vax 4 m (Australia); Hipracox Broilers (Spain); Immuner gel coc (Argentina); Livacox Q (it is a quadrivalent live attenuated vaccine for breeders and layers against *E*. *acervullina*, *E*. *tenella*, *Eimeria necatrix*, *E*. *maxima)* and Livacox T (Czech Republic) [17]. Mixed vaccines consisting of precocious strain of *E*. *tenella* and non-attenuated strains of *E*. *acervullina* and *E*. *maxima* are produced named as Paracox5 and Paracox8 (UK) and Supercox (China), it is a mixed strain vaccine consisting of precocious strain of *E*. *tenella* and non-attenuated strains of *E. acervulina* and *E*. *maxima* [2,17]. The commercial success of this type of vaccine lies primarily in breeder and layer flocks where anti-coccidial drugs have been banned to prevent the carryover of drugs into eggs and meat [15].

b) Vaccines based on drug-abbreviated infections

This vaccination technique was developed in 1970 and involves an infection and treatment strategy. Hemoprotozoan parasite infections are not self-limiting, and parasites can proliferate continuously in the blood stages if not checked by the immune response or drug treatment. Solid, sterile immunity develops after primary infection, and vaccination of cattle by infection with pathogenic wild-type *Theileria parva* followed by drug treatment (long-acting tetracyclines) has been used for many years to control East Coast fever [15]. In India, live sporozoite vaccine of *Theileria annulata* from infected ticks accompanied by chemo immunoprophylaxis with tetracycline or buparvaquone is formulated [18]. Protection is thought to be conferred mainly by cell-mediated immunity, more specifically, CD8-cytotoxic T-lymphocytes, against the intracellular schizont [19].

c) Vaccines based on infections with parasites with a truncated life cycle

Cysts are produced by several protozoans within the host that are a persistent source of infection when eaten by other animals. These cysts can also cause re-infection when the immune system is compromised or can be reactivated during pregnancy, causing congenital disease, and abortion. *Toxoplasma gondii* infects a wide variety of hosts, including humans and is the major cause of abortion in sheep and goats. As immunity to primary infection develops, the intracellularly replicating tachyzoites become encysted in a dormant stage (zoitocysts), which can persist for several years, containing hundreds of infective bradyzoites. *T. gondii* parasites that were continuously passage in mice to produce diagnostic antigens were later found to have lost their ability to form cysts. The S-48 strain of *T*. *gondii* is used as a live vaccine with a trade name of Toxovax and is currently only commercial protozoan vaccine for toxoplasmosis. S-48 is a laboratory adapted strain that has lost the ability to differentiate into bradyzoites [20]. The “incomplete” S48 strain of *T. gondii* forms the basis of a commercial vaccine conferring long-lasting immunity (approximately 18 months) of susceptible ewes against *Toxoplasma*-induced abortion when administered prior to mating [21]. As S-48 strain undergoes limited multiplication within host animal and thus stimulates an appropriate cell-mediated immune response involving CD4+, CD8+ T cells, and IFN, therefore not permitting the parasite to persist. This vaccine primes the immune system so as to prevent the spread of parasite within lymphatic systems and thus limiting the spread to the circulation of host. The vaccine is normally administered prior to mating and there is a meat and milk withdrawal period of 6 weeks following vaccination [18].

d) Vaccines based on virulence-attenuated strains

Attenuated cell line vaccines against various blood protozoans like *Babesia bovis*, *Babesia bigemina*, *T*. *annulata*, *Anaplasma* spp. has been used successfully in a number of countries, e.g. Isarel, Iran, Morocco, Tunisia, India, China, etc. As reviewed by [22,23], attenuated, but still immunogenic piroplasms of *B*. *boviss* and *B*. *bigemina* obtained after continuous passaging in splenectomized calves, live vaccines using infected blood collected from acute infections of splenectomized calves were developed in Australia several decades ago and are still used in most countries to protect against babesiosis. This vaccine sometimes supplemented with *Anaplasma centrale* infected blood where *Anaplasma marginale* is enzootic. Attenuated cell line vaccine of *T*. *annulata* are used in India and protection is engendered by the attenuated schizont vaccine which has been evaluated by laboratory challenge with live infected ticks or with ground up tick sporozoites inoculated through syringe passage. The results of these challenges vary from no clinical response at all to mild transient clinical reaction with parasitemia. This attenuated schizont vaccine was commercialized under the trade name of “RakshaVac T” and is produced and marketed by Indian Immunological at Hyderabad, India [18]. A continuous exposure to natural tick infections is generally required to ensure continuous and long-lasting immunity through these virulence attenuated vaccines. To increase shelf life and safety frozen-blood vaccines stored in liquid nitrogen using either dimethyl sulfoxide or glycerol as the cryoprotectant are produced [15].

## Killed and Subunit Protozoan Vaccines

Several inactivated vaccines consisting of crude whole organisms or, more recently, defined antigenic structures are available and are registered for companion animal market. In general, these vaccines are not as effective as live organisms, but can mitigate disease or transmission to various degrees. They may form the basis for the development of recombinant vaccines. The challenge in developing effective killed vaccine is to select an appropriate parasitic antigen and delivers it to the immune system in such a form that it should have the capacity to induce effective innate and adaptive immune response [18].

### Killed vaccines

*Neospora caninum* is a major cause of abortion in cattle (intermediate host). A crude *N*. *caninum* vaccine consisting of inactivated *N*. *caninum* tachyzoites with an adjuvant available in the United States to aid in the reduction of *N*. *caninum* induced abortion in healthy pregnant cattle and prevents the transmission of the parasite to calves *in-utero*. Neogaurd is the trade name of this vaccine [24,25]. *Giardia intestinalis* is an enteric parasite of many animal species and can cause severe GI disease in young and immune compromised individuals. Giardia Vax^®^ a commercial vaccine has been licensed for use in dogs and cats in USA and it is commercialized to significantly reduce the incidence, severity, and duration of cyst shedding. The vaccine consists of a crude preparation of disrupted, axenically cultured *G*. *intestinalis* trophozoites (sheep isolate) [26,27]. Marsh [28], evaluated and proposed a vaccine that alleviates equine protozoal myeloencephalitis caused by *Sarcocystis neurona*. Epm Vaccine consists of *in-vitro* cultured merozoites, which are chemically inactivated, originally obtained from the spinal cord of a horse, and mixed with an appropriate adjuvant. Trichguard vaccine is a killed vaccine containing axenic culture in broth containing killed trophozoites in adjuvant against *Tritrichomonas foetus* for cattle [29].

### Subunit and recombinant vaccines

Subunit and recombinant vaccine consist of certain key molecules that manipulate the host immune response for their survival [30] and thus by negating the function of these molecules prevent the establishment of parasite in the host. Substantial approaches have been achieved in recent years for the development of subunit and recombinant vaccines [31-33].

*Theileria* sporozoite and schizont antigens have been identified for development of subunit vaccines. Sporozoite antigens are surface antigens (SAGs) of both *Theileria parva* (p67) and *T*. *annulata*. Recombinant immunodominant sporozoite gene for *T*. *annulata*, i.e., SPAG-1 antigen has also been identified and tested, but all these antigens does not provide protection in a required level [18]. Two subunit vaccines against canine babesiosis have been developed. Both vaccines consist of soluble parasite antigens (SPA) that are released into the culture supernatant by *in-vitro* -cultured parasites, combined with adjuvant. The first vaccine is, Pirodog^®^, contains SPA from *Brucella canis* cultures only [34], whereas recently developed is NobivacPiro^®^ which contains SPA from *B*. *canis* and *Babesia rossi* in an attempt to broaden the strain-specific immunity. The protective effect of this vaccine seems to be based on the antibody-dependent neutralization of a soluble parasite substance [35]. A killed vaccine approach was also formulated for *T*. *gondii* by using different recombinant proteins like membrane-associated SAGs, dense granules antigens rhoptry proteins, and micronemal proteins [14].

Subunit vaccine against *E*. *maxima* is commercialized in Israel and it consists of two purified gametocyte antigens, i.e., GAM56 and GAM 82. This vaccine results in the induction of maternal immunity which is boosted by natural infection. Immunized laying hens transfer maternal antibody IgY to chicks [17,36].

“Leishmune” a subunit vaccine has been registered in Brazil against visceral leishmaniosis. It consists of a glycoprotein complex isolated from *Leishmania donovani*, fucose mannose ligand, and a saponin adjuvant. This vaccine is also useful in blocking transmission and thus is able to control zoonotic visceral leishmaniosis [18]. CaniLeish provisionally licensed in parts of Europe and contain excreted and secreted proteins of *Leishmania infantum* [37]. Lemesre *et al*. [38], showed 92% efficacy of this vaccine over a period of 2 years. Leish-Tecis licensed in Brazil since 2007 and is only recombinant vaccine for protozoa containing *Leishmania* amastigote antigen (A2) purified from transfected *Escherichia coli*.

## Helminth Vaccines

There are three different families of helminthes nematodes (roundworms), trematodes (flatworms), and cestodes (tapeworms). These are multicellular parasites having genetic complexity, and there physical size, cannot be internalized by phagocytic cells of the immune system or killed by classical cytotoxic T cells. Hence, immune system has to develop a new mechanism referred to as the Type 2 or allergic-type immune response, typified by the recruitment and activation of potent effector leukocytes, mast cells, and eosinophils [39,40].

### Vaccines against trematodes

The most important disease in terms of geographical distribution and economic losses to farmers is Fasciolosis caused by *Fasciola hepatica* and *F*. *gigantica* [41]. According to Raina [41], no viable vaccine are present because of the variable efficacy between animal species and the protection level to date is <80%. Limited number of candidate antigen are known for *Fasciola* spp. and no viable vaccine has been developed with protection level more than 80%. Various vaccine candidates have been identified as key targets for vaccination against *Fasciolosis*. These are Cathepsin-B and Cathepsin-L, which are present in excretory and secretory materials of *Fasciola* spp., these are then expressed in yeast and used as a multivalent vaccine in a rat model. Vaccination of Friesian cattle with recombinant *F*. *hepatica* cathepsin L-1 protease formulated in Montanide 70 VG and 206 VG was done and the animals were exposed to fluke contaminated pasture for 13 weeks and there was a reduction of 48.2% in fluke burden as compared to control and non-vaccinated groups. Other vaccine candidates are leucine amino-peptidase (LAP) present in bacteria, plants, and animals and are novel drug targets and vaccine candidates in parasitic infections [42]. Partial purification and characterization of LAP from *F*. *hepatica* and vaccination in sheep has shown 89% protection. Piacenza [42] formulated a cocktail of LAP, CL-1, and CL-2 which gave 78% protection. However, production of viable vaccine is difficult as the parasite has the ability to target similar host pathways and has evolved its own strategy to confound host defenses [43].

### Vaccines against cestodes

Vaccines developed against cestodes are recombinant vaccines, which are based on antigens of the parasite stage that adhere to the gut wall and induce immune responses that interfere with successful attachment. In 1990s, effective recombinant vaccines were developed against the cestodes *Taenia ovis*, *Taenia saginata*, *Taenia solium*, and *Echinococcus granulosus* [12]. To date, only the vaccine against the cestode *T*. *ovis* has been registered in Australia and New Zealand, but has not been marketed. The antigens are 45 W, TSOL-18, TSOL-45 and EG-95 for *T*. *ovis*, *T*. *solium*, *E*. *granulosus*, respectively all these are Oncosphere proteins and interferes with attachment [12,44].

### Vaccines against nematodes

There are two strategies for vaccine development against GI nematodes - attenuated vaccines and hidden antigen approach [45].

## Attenuated Vaccines

The first and most effective vaccine against lungworms was a ×1000 - an irradiated larval vaccine against *Dictyocaulus viviparous* infection in cattle named “DICTOL” and is commercially available and marketed in Europe. The vaccine was developed [46] and contains irradiated L3-larvae that do not mature to adult worms. It is administered in two doses of attenuated larvae and induced up to 98% protection against challenge infection. In India, vaccine against sheep lungworm *Dictyocaulus filaria* was developed using a similar approach of 50 k R gamma irradiated attenuated L3 larvae and was named “DIFIL” [47]. A similar approach was used to develop a vaccine against the canine intestinal nematode *Ancylostoma caninum* [48], but it was observed that irradiation-attenuated larval vaccines developed against GI nematodes did not protect young, susceptible stock against infection and were, therefore, never commercialized [49]. Moreover, larvae must be harvested from the manure of infected animals, so these vaccines are difficult to produce too.

### Vaccines based on hidden antigens

Hidden antigens are integral membrane proteins associated with the gut of GI nematodes. Hidden antigens discovered against *H*. *contortus* are H 11, H-gal-P, cysteine proteases, and Enolase [45]. The principle of immunization is that recombinant hidden antigens molecules induce a high level of antibody which cross-reacted with native antigen molecules.

## Ecto-parasite Vaccines

Ectoparasitic arthropods would seem to be the ultimate challenge in vaccine development, as they not only are large and complex but also spend most of their life outside or on the surface of the host. Of all the ectoparasites ticks poses a serious threat in livestock industries. They are obligate hematophagous arthropods that parasitize every vertebrate and are responsible for transmission of various tick-borne diseases and can also cause severe toxic conditions, paralysis, and allergy [50]. Vaccine against the cattle tick, *Rhiphicepahalus* (*Boophilus*) *microplus*, is a recombinant vaccine based on a protein (Bm86) found in the tick at the surface of the gut wall. It was first introduced commercially in Australia in 1994 (TickGUARD) [51]. This vaccine is unique in that it is not based on natural antigens recognized by the immune system during infection, but takes advantage of the ferocious blood-feeding habits of the tick. Vaccination stimulates the production of high antibody levels in cattle against a tick gut membrane-bound protein, Bm86, using a recombinant protein in a potent adjuvant. These antibodies bind to the tick’s gut surface when taking a blood meal, causing the rupture of the gut wall, and tick death [52]. The vaccine induces significant levels of protection against tick infestation and, in some cases, against tick-borne diseases. However, antibody levels are not boosted by infection and need to be maintained at high levels by repeated immunization. Use of vaccine in conjunction with drug administration limits its practical and commercial use. Moreover, the presence of a tick immunoglobulin excretion system hampers the effectiveness of this vaccine approach in other ticks [53]. Another vaccine for tick Gavac which is a Cuban vaccine contain recombinant *Rhiphicepahalus* (*Boophilus*) *miroplus* Bm86 gut antigen expressed in *Pichia pastoris*. This is 99% effective against *Rhiphicepahalus* (*Boophilus*) *microplus* and is 100% effective against *Rhiphicepahalus* (*Boophilus*) *annulatus* [53]. Summarized table depicting various commercial antiparasitic vaccines, which are available in various parts of world are given below in Table-1.

**Table-1:** Antiparasitic vaccines commercially produced.

Parasite	Host	Type of vaccine	Comments	References
*Eimeria* spp.	Poultry	Live virulent	Non attenuated low doses of infective oocyst.	[15,17]
*Eimeria* spp.	Poultry	Attenuated for precocity	Infection immunity. Attenuation achieved by several passages in chicken embryos or by using the precocious lines	[17]
*E. maxima*	Poultry	Subunit vaccine of gametocyte antigen	Consist of two GAM 56 and GAM purified gametocyte antigens. Results in induction of maternal immunity	[36]
*T. gondii*	Sheep	Attenuated for truncated life cycle.	Live attenuated S - 48 strain to reduce congenital infection in ewes. Originally isolated from an aborted ovine fetus in New Zealand	[20,21]
*N. caninum*	Cattle	Killed vaccine	Killed tachyzoites. Reduces abortion	[24,25]
*T. annulata*	Cattle	Attenuated cell line vaccine	Consist of attenuated schizont which has been evaluated by laboratory challenge with live infected ticks or with GUTS inoculated through syringe passage	[15,18]
*T. parva*	Cattle	Non attenuated live vaccine	Immunity induced by non-attenuated wild-type parasite’s sporozoites. Infection is controlled by chemotherapy	[15]
*B. bovis*, and *B. bigemina*	Cattle	Attenuated vaccine	Consist of immunogenic merozoites of *B. bovis* and *B. bigemina* obtained by repeated passage through splenectomized calves	[22,23]
*B. canis*	Dog	Subunit vaccine	Consist of SPA and gives protection by antibody-dependent neutralization of a soluble parasite substance	[34,35]
*G. duodenalis*	Dogs	Killed vaccine	Reduces incidence, severity, duration of cyst shedding, consists of a crude preparation of disrupted, axenically cultured *G. intestinalis* trophozoites	[26]
*T. ovis*	Sheep	Subunit recombinant vaccine	Candidate antigen: 45 W an oncosphere protein Interferes with attachment to gut wall	[12,44]
*Dictyocaulus viviparous* *Dictyocaulus filaria*	Cattle	Live irradiated vaccine	Contains irradiated L3-larvae that do not mature to adult worms	[46,47]
*Rhiphicepahalus (Boophilus) microplus*	Cattle	Recombinant vaccine	Consist of protein (Bm86) found in the tick at the surface of the gut wall	[50,51]

*E. maxima=Eimeria maxima, T. gondii=Toxoplasma gondii, N. caninum=Neospora caninum, T. annulata=Theleria annulata, T. parva=Theleria parva, B. bovis=Babesia bovis, B. bigemina=Babesia bigemina, B. canis: Babesia canis, T. ovis: Taenia ovis, G. intestinalis=Giardia intestinalis*, SPA=Soluble parasite antigen, GUTS=Ground up tick sporozoitesk, *G. duodenalis=Giardia duodenalis*

## Edible Vaccines

The edible vaccines are a novel approach for sustainable development of vaccines for prophylaxis. According to [54,55] vaccine, genes are expressed in edible plants, which are consumed by animals. As a major alternative, plants are emerging as a promising system to express and manufacture a wide range of functionally active parasitic antigens [56]. Plants like alfalfa, tobacco, lettuce, potato, maize rice, banana, wheat, corn carrots, peanuts, and soybeans have been studied for vaccine production. The major advantages of edible vaccines are that once plants are created there seeds can be used for stored for future use, these are cheaper to manufacture and there is no need of purification and processing as the final product are edible [2].

With so many advantages the drawbacks of edible vaccines are there genetic variation, batch to batch variation unexpression of vaccine candidates in plants, poor immunogenicity of certain vaccines in the host, not suitable for all types of animals, and there can be development of oral tolerance to the antigens [2].

## Conclusion

Future of developing vaccines for control of parasitic infections in animals is bright and is most sustainable and economical approach. There is need of maintaining synergism between parasitic vaccines and anti-parasitic drugs as this will help in the reduction of dependence on anti-parasitic drugs, thus decreasing the development of resistance. Traditional methods of control of parasites like chemotherapy, grazing management, biological control of parasites can be used in an integrated pattern for efficient control but are not feasible economically in many countries including India. Vaccines offer long-lived immunity that can be repeatedly boosted when animals are exposed to natural infections. The first most effective vaccine against a parasitic disease was an irradiated larval vaccine against *D*. *viviparous* infection in cattle named DICTOL and on the same principle of irradiation first parasitic vaccine in India was against *Dictyocaulus filaria* (Lungworm of sheep) named DIFIL (1981). In the field of helminth vaccines, various subunit vaccines have been developed using various candidate antigens but due to their evasion mechanism from the host immunity development of vaccines for helminthes is still in progress. Various live vaccines are produced against protozoan parasites and are licensed and commercialized to large scale in many countries including India, but these vaccines poses a risk of reversion of non-virulent or attenuated strain to virulent strain in immune-compromised animal leading to failure of vaccination and live vaccine also have short shelf life and need cold chain to deliver. In spite of these drawbacks live schizont vaccine of *T*. *annulata* is a successful commercialized vaccine in India with a brand name of RakshaVac-T formulated by Indian Immunologicals, Hyderabad. Vaccines against zoonotic and foodborne parasites (*T*. *ovis*, *T*. *solium*, *Echinococcus multilocularis*, poultry *Coccidia* etc.) are important as they are aimed at reducing risk for the consumer and are also responsible for increase in production. A number of vaccines have been formulated and commercialized in various parts of the world regarding this aspect like Coccivac B, Coccivac D, Immunocox C1, Paracox, etc. vaccine against *T*. *ovis* containing 45 W subunit antigen. There are many hurdles in vaccine development due to immune evasion capacity of parasites, antigenic diversity but advances in field of molecular biology, proteomics, immunology, and recombinant technologies have provided a way for the development of vaccines for animal parasites.

## Authors’ Contributions

NS prepared the initial version of the manuscript. VS helped in the collection of literature and KPS done the scientific and technical corrections.
